# Hygiene practices during clinical training: knowledge, attitudes and practice among a cohort of South Asian Medical students

**DOI:** 10.1186/s12909-019-1582-2

**Published:** 2019-05-21

**Authors:** U. Jayarajah, A. S. Athapathu, B. A. A. J. Jayawardane, S. Prasanth, S. N. Seneviratne

**Affiliations:** Department of Paediatrics, Faculty of Medicine, University of Colombo, P.O. Box 271, Kynsey Road, Colombo 8, Western Province Sri Lanka

**Keywords:** Knowledge, Attitude, Hygiene practices, Medical students, Hand hygiene, Equipment, Attire, Clinical coat

## Abstract

**Background:**

Proper hygienic practices are important in preventing nosocomial infection. This study aimed to determine knowledge, attitudes and practices (KAP) on hand, attire and equipment hygiene during clinical training among medical students at a State Medical Institution in Sri Lanka.

**Methods:**

This cross-sectional study was conducted among 3rd, 4th and final (5th) year medical students of the Faculty of Medicine, University of Colombo, who had undergone at least 6 months of clinical training. KAP on hand hygiene (HH), attire hygiene (AH) and equipment hygiene (EH) were assessed using a pre-tested, self-administered questionnaire with a Likert-type scale. KAP scores were graded as follows: good ≥75; moderate:74.9–50; unsatisfactory:49.9–25; poor:< 25%. KAP based on duration of training and gender were compared using independent samples t-tests.

**Results:**

Three hundred thirty-three students participated (mean age 24 ± 1.1 years, male: female = 1: 1.2). Combined KAP scores on hand and attire hygiene were moderate (HH:73%, AH:65%) while equipment hygiene was unsatisfactory (EH:47%). Senior students (5th year) had higher combined KAP and knowledge (K) on hand hygiene (HH KAP 75% vs. 72%, *p* = 0.01; K:72% vs. 67%, *p* = 0.001) and equipment hygiene (EH KAP 50% vs. 44%, *p* = 0.001; K:47% vs. 35%, *p* = 0.001) compared to junior students (3rd/ 4th years). However, they had lower KAP and P scores on attire hygiene (AH KAP 63% vs. 67%, *p* = 0.006; P:60% vs. 67%, *p* = 0.004). Female students had better AH compared to male students (KAP:67% vs. 64% *p* = 0.01; K 71% vs. 66%, *p* = 0.048; P:66% vs. 62%, *p* = 0.05).

**Conclusions:**

Overall, hand hygiene was moderate among medical students and improved with progression of training. Attire hygiene was also graded as moderate but to a lesser extent compared to hand hygiene, lower in males, and declined over time, indicating need for better reinforcement of attire hygiene practices with progression of clinical training. Equipment hygiene was unsatisfactory among most medical students and thus needs to be highlighted as a potential area to be improved during clinical training. This study suggests that knowledge, attitudes and practices on equipment and attire hygiene among medical students was less satisfactory and needs to receive greater emphasis during medical clinical training.

**Electronic supplementary material:**

The online version of this article (10.1186/s12909-019-1582-2) contains supplementary material, which is available to authorized users.

## Background

Healthcare associated infections are one of the commonest causes of morbidity in both developed and developing nations [1]. The burden is greater in developing regions with limited resources, such as South Asia [1]. Spread of infection can be prevented by maintaining good hygiene practices.

In clinical practice hand hygiene is the simplest and cost effective way to reduce the incidence of health care associated infection transmission [2]. However, while evidence based concepts and strategies are in place to improve hand hygiene, implementation appears to be low [3]. Furthermore, unsatisfactory hygiene practices in relation to attire such as clinical coats and medical equipment such as stethoscopes, tapes, and knee hammers is also associated with the spread of healthcare associated infections [4–6]. Healthcare workers clinical coats especially can harbor potentially harmful pathogenic organisms [7]. The Society for Healthcare Epidemiology of America recommends that medical personnel including medical students should have at least two clinical coats, and these coats should be washed at least once a week, and when there is visible dirt [6]. Short sleeved attire, and avoidance of watches and hand accessories to minimize harbouring and transmission of pathogens is also recommended [6]. Equipment such as stethoscopes and tapes which are used frequently in clinical practice and are placed directly in contact with the patients’ skin are also subject to contamination. Transmission of pathogens can occur easily via these equipment resulting in healthcare associated infections [8]. Thus, implementing disinfection hygiene maintenance frequently is recommended for prevention.

Possible factors leading to poor adherence with hygiene practices include lack of knowledge regarding recommendations of hygiene practices in the clinical setting, lack of time due to the busy work schedule, lack of readily available facilities, irritant contact dermatitis due to frequent exposure to soap and disinfectants and failure of higher authorities to implement hygiene practices as an important institutional priority [9].

Importantly, previous studies indicate that medical students may have unsatisfactory knowledge and practices related to hygiene [10–13]. As medical students are frequent regular visitors to hospital wards during their clinical training years, poor hygiene practices among them can increase spread of infections. Further, their training and practices during the medical student period can reflect their future hygiene practices as healthcare providers. Assessing hygiene practices among medical students undergoing clinical training, and determining their adequacy is therefore of considerable importance. Identifying problem areas and taking necessary interventions to correct them will be beneficial not only to the trainees, but will also have a greater bearing on preventing spread of nosocomial infection in the future, as these students progress to become medical practitioners and role models to other health care workers long-term. There is however, a paucity of data regarding hygienic practices among medical students in the region. The aim of this study was to assess knowledge, attitudes and practices regarding hand hygiene, attire hygiene and equipment hygiene among South Asian Medical students undergoing clinical training at the Faculty of Medicine, University of Colombo, Sri Lanka. Furthermore, level of knowledge, attitudes and practices were compared between students of different stages of clinical training and gender.

## Methods

A descriptive cross-sectional study was carried out at Faculty of Medicine, University of Colombo between August and September 2015. Medical students, who had completed at least 6 months of clinical training, were eligible to participate in the study. Informed consent was obtained from each participant. Ethical clearance was obtained from the Ethics Review Committee of the Faculty of Medicine, University of Colombo.

Clinical training refers to teaching and hands-on training of medical students conducted in actual hospital settings. In this study setting, clinical training commences in the 3rd year of the curriculum. During the 3rd and 4th years of the medical curriculum, students have ½ day of clinical training and ½ day of classroom-based teaching daily. During the fifth (final) year of the medical curriculum, approximately 90% of the teaching/ training is hospital-based clinical training.

A pre-tested semi-structured self-administered questionnaire (Additional file 1) was used to assess knowledge, attitudes and practices of participants regarding hand, attire and equipment hygiene. Selection of items were based on previous similar studies and guidelines on prevention of healthcare associated infection [11, 14, 15]. These items were pooled and practically relevant and important items were selected by the investigators to prepare the preliminary questionnaire. The questions were then reviewed and revised as necessary for accuracy, construction problems, and grammar. Both pretested scales and open ended questions were used to assess the practice. Observations were not used to assess practice.

The questionnaire (Additional file 1) consisted of separate sections on assessment of knowledge (K) attitudes (A) and practices (P) in relation to hand hygiene (HH), attire hygiene (AH) and equipment hygiene (EH). It was modelled upon previous studies on hygienic practices [11, 14] and standard recommendation guidelines regarding hygiene practices to prevent health-care associated infections [15]. At the end of the questionnaire, students were provided with additional space to note down reasons for suboptimal practice and suggestions for improving hygienic practices.

Knowledge (K) in each component (HH, AH and EH) was assessed using 20 questions with 3 responses (yes /no/unsure). Attitude (A) was measured using 20 questions in which respondents were asked to choose a single option on a 5 point Likert scale where 0 = strongly disagree and 4 = strongly agree. Practice (P) was assessed using a 5 point Likert scale where 0 = never and 4 = always. Final score in each area was calculated by adding up the points and converting to a percent score (range 0 to 100). The scores were graded assigning cut-off values used by similar studies (good ≥75, moderate 50–74.9, unsatisfactory 25–49.9, poor < 25%) [11, 14]. Combined (KAP) scores in each component were obtained by adding the K, A and P scores and calculating the average.

Internal consistency of the modified questionnaire was measured using Cronbach’s alpha. Cronbach’s alpha values of the main scales for knowledge attitudes and practice ranged from 0.728 to 0.786 and those for subscales measuring for hand, attire and equipment hygiene ranged from 0.629 to 0.843 showing acceptable internal consistency. The Kaiser-Meyer-Olkin test (KMO) which is a measure of sampling adequacy was 0.86 which indicated an adequate sample size and Bartlett’s test of sphericity was highly significant (*p* < 0.001).

The pre-final version of the questionnaire was pilot tested on 30 students. After completing the questionnaire, each respondent was requested to elaborate what they thought each questionnaire item and their corresponding response meant. These students were not included in the final analysis. Following pretesting the questionnaire, no major adjustments were made but finer correction were made to improve the clarity so that the intended meaning was retained.

Data processing and analysis was done using SPSS statistical software ver.20 and results were expressed as frequency and percentages. Comparison of hygiene practices in relation to gender and stage of clinical training was done using independent samples t-test. Spearman’s Rank Correlation tests were used to determine strength of associations between individual and combined scores on knowledge, attitudes and practices overall and with regard to each component of hygiene (HH, AH and EH). A *p* value of < 0.05 was considered as statistically significant.

## Results

From a total of 440 medical students who were eligible for the study, 333 (75.7%) students participated in the study. Mean participant age was 24.4 ± 1.1 years, with a male to female ratio of 1: 1.12. Of the participants, 142 (42.6%) students were in the final year.

**Fig. 1:**
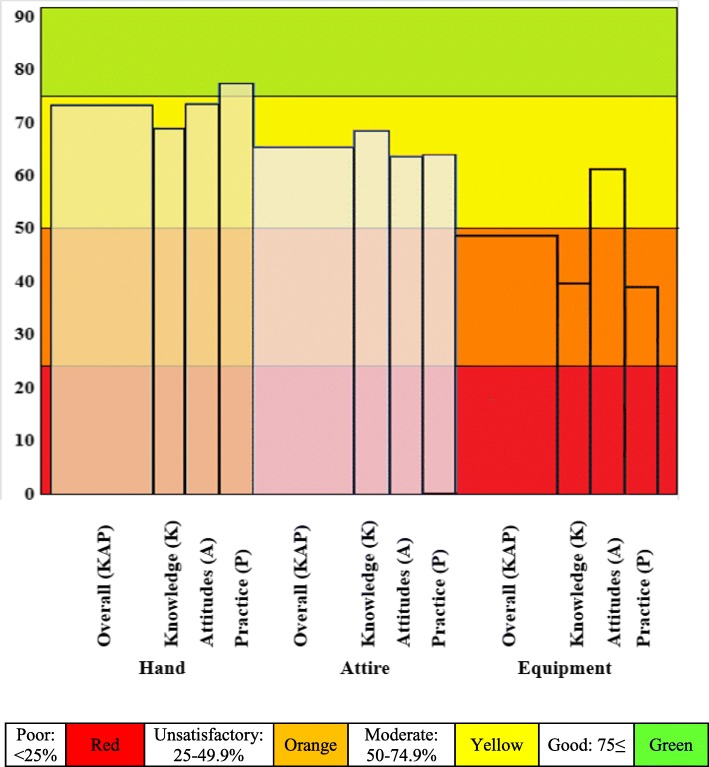
Mean scores on knowledge (K), attitudes (A) and practices (P) on hand, attire and equipment hygiene among medical students

Overall, medical students scored best on hand hygiene, less on attire hygiene, and worst on equipment hygiene (Total KAP scores 73, 65 and 47% respectively, *p* < 0.001). On grading, combined KAP scores on hand and attire hygiene were moderate while equipment hygiene was unsatisfactory (Fig. 1). When considering individual K, A and P component scores, notably hand hygiene P was good (77%), and equipment hygiene K and P scores were unsatisfactory (40 and 39% respectively), while all other aspects assessed were in the moderate range (Fig. 1).

When considering the stage of medical training, final year medical students scored higher on hand and equipment hygiene compared to their juniors, (higher combined KAP and K scores, and tendency to higher P scores, see Table 1) and achieved a ‘good’ combined KAP score for hand hygiene. Hand hygiene P score remained good at both stages of clinical training (Table 1). Further, final year students achieved a ‘satisfactory’ total KAP score for equipment hygiene (50.1%). However, K and P scores for equipment hygiene remained at an ‘unsatisfactory’ level as in the juniors. Notably, senior students scored lower than junior students on attire hygiene (lower total KAP and P scores, see Table 1).

**Table 1:**
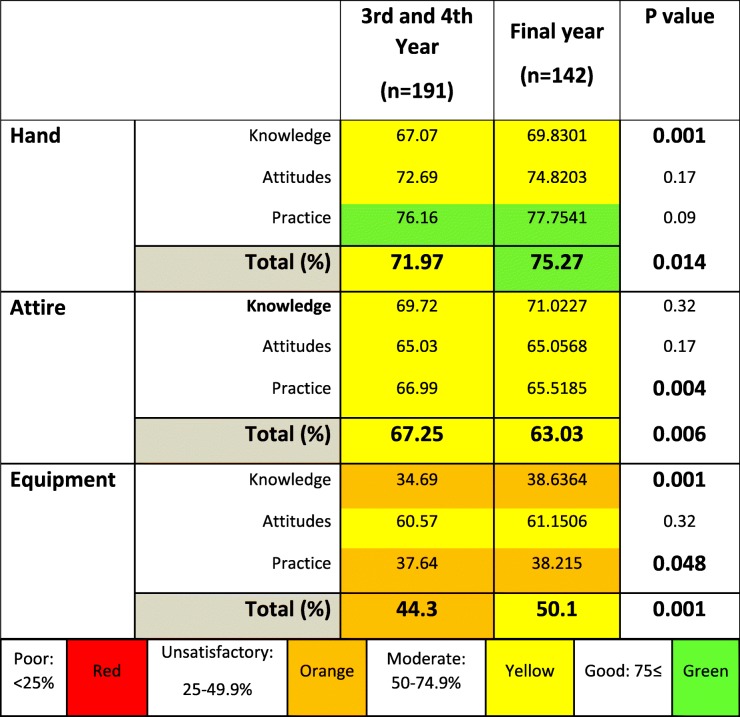
Comparison of knowledge, attitudes and practice scores on hand, attire and equipment hygiene in relation to year of medical training

When considering gender-based differences in hygienic practices among medical students, female students showed better attire hygiene (Table 2). No differences were seen in relation to hand and equipment hygiene (Table 2).

**Table 2:**
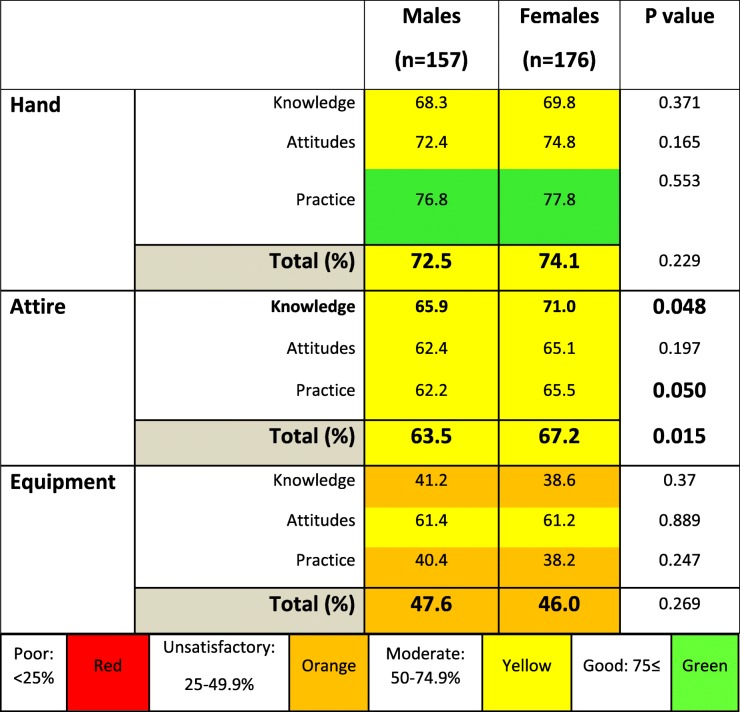
Comparison of knowledge, attitudes and practice scores on hand, attire and quipment hygiene between male and female medical students

When analysing reasons for suboptimal hygiene practices: the majority (*n* = 233, 78%) said that they were not aware of the proper recommendations/techniques for equipment and attire hygiene, and therefore lack of knowledge was the main reason for unsatisfactory attire and equipment hygiene. Further, 32% (*n* = 105) stated that ward-based facilities for hand hygiene were not adequate.

There were positive associations between attitude and practice; and knowledge and practice; overall and in all three individual components (HH, AH and EH). Overall knowledge was weakly positively associated with practice (rho = 0.35, *p* < 0.001), while the association between overall attitudes and practice was moderately positive. (rho = 0.55, *p* < 0.001). When considering hand hygiene, the association between practice and knowledge continued to be weak (rho = 0.34, *p* < 0.001) but a strong positive association was noted between attitudes and practice (rho = 0.62, *p* < 0.001).

## Discussion

### Summary of results

In this study, knowledge, attitudes and practices regarding hand and attire hygiene among medical students undergoing ward-based clinical training were graded as moderate while knowledge and practices on equipment hygiene were unsatisfactory. Hand and equipment hygiene were better among final (fifth) year students, but attire hygiene was lower in comparison to juniors in their third and fourth years of medical training. This may reflect on the busy clinical training resulting in lack of time for cleaning their attire. Gender-based differences were noted only in attire hygiene, with female students showing better knowledge and practices compared to male medical students. The major reason given for not adhering to attire and equipment hygiene was lack of knowledge on recommendations, while the main reason for not adhering to hand hygiene was suboptimal facilities for hand washing.

### Comparison of results with other similar studies

In the present study, we focussed on hand hygiene, equipment hygiene and attire hygiene. However, most previous studies conducted in Sri Lanka and elsewhere among medical students and health care workers have focussed only/ mainly on hand hygiene. Several studies have shown that although medical students had adequate knowledge on hand hygiene, there were significant deficits in the practice of hand hygiene [11–13, 16]. A study by Ariyarathne et al in a group of Sri Lankan medical students showed that although 83% had satisfactory (scores above 50%) knowledge on hand hygiene, attitudes were satisfactory in only 13% and practices in 20% [11]. In another study conducted by Arthi et al. in a Medical school in South India, nearly 85% of the medical students failed to adhere to proper hand hygiene practices although knowledge related to hand hygiene was good [16]. Findings compatible with our study has been reported among Saudi Arabian medical students, with 70% showing adherence to proper hand hygiene techniques (18).

A study by Barroso et al. among medical students and residents at Stanford University School of Medicine found that knowledge was not a significant predictor of behavior, while a favorable working environment and observing attending physicians with good hand hygiene practices were reported to be effective strategies influencing practice [17]. A descriptive, cross-sectional study among Saudi nursing students showed that 68.7, 29.8, and 1.5% of the respondents had moderate, good, and poor practice of hand hygiene, respectively. Those who were in a lower academic level of nursing education and those who attended seminars related to hygiene had better hand hygiene practices [18]. A descriptive cross-sectional study of 137 clinical students of Bayero University Kano showed that 62.8% of students adhered to the principles of hand-washing in their clinical postings, but only 52.6% students washed their hands before handling patients, although a majority 130 (94.9%) washed their hands after handling patients [19]. A cross sectional study among Italian nursing and medical students attending clinical wards for practical training showed that knowledge and practice were significantly higher in nursing compared to medical students [20].

In general, hygiene practices in clinical settings in the region and other countries have been shown to be unsatisfactory. Thus, measures to improve hygiene practices have been proposed in the above mentioned studies. Furthermore, it is notable that none of these other studies mentioned assessed attire and equipment hygiene. As equipment and attire related hygiene practices were lower than hand hygiene in our study, it is possible that attire and equipment hygiene may have been quite unsatisfactory in the other studies, if assessed.

In studies reporting poor hand hygiene practices, participants have stated lack of time, emergencies and forgetfulness as the main reasons for not practicing hand hygiene. In our study, students had moderate knowledge and attitudes towards hand hygiene, and their hand hygiene practices was good. Further, the main reason mentioned as a factor for not practising hand hygiene was lack of facilities for hand washing (32%). This suggests indirectly, that these students had actively looked for the facilities and found them lacking. Based on the results of this study, and those reporting a lack of association between knowledge and practice of (hand) hygiene, we postulate that better attitudes together with better knowledge on hand hygiene could have led to greater adherence with hand hygiene practices seen in this study.

### Implications and recommendations

In this study, majority of medical students were unaware of recommendations to clean their equipment and attire. This suggests that greater emphasis should be given to improve knowledge and awareness on attire and equipment hygiene among medical students. Although knowledge and practice of equipment hygiene were unsatisfactory, attitude scores were moderate, indicating that improving students’ knowledge may lead to an improvement in equipment hygiene [21].

Hence it is necessary to conduct training programmes to bridge the gap in knowledge, emphasising on recommendations for equipment and attire hygiene. Ensuring continuous availability and easy accessibility for facilities for maintaining hand, equipment and attire hygiene in the clinical setting could also encourage better practice. Previous studies have shown that regular hand hygiene training sessions, displaying posters and encouraging peers have led to improved compliance with hygiene practices [21, 22]. In our study, hygienic practice in relation to three aspects assessed (HH, AH and EH) showed positive associations with both knowledge and attitudes, with stronger associations between attitudes and practice, especially for hand hygiene. Thus, those with higher scores on knowledge and attitudes had higher scores in practice, with greater association with attitudes than knowledge. However, the correlation presented was between low and moderate, which means that the amount of variance explained was quite low. Thus, improving knowledge may not necessarily change their practices. However, this is a very interesting question for future research: understanding why students who possess correct knowledge on hygiene practices do not use it in practice.

We propose the following refinements in the curriculum to improve the hygiene practices. Efforts should be taken to improve attitudes of students towards ward-based hygiene practices, as well as imparting the necessary knowledge. This may possibly be done through positive role modelling and workplace based assessments etc. Further, there appears to be a lack of knowledge among medical students in relation to equipment hygiene, and this deficit in knowledge needs to be addressed. Hygiene related training sessions may need to be conducted more frequently for medical students with continuous monitoring and performance feedback to encourage them to adhere to proper hygiene practices for equipment and attire hygiene and maintain good standards in hand hygiene.

## Conclusion

Overall, hand hygiene was moderate among medical students and increased with progression of training, but could be improved further to reach better standards by increasing facilities for handwashing. Attire hygiene was moderate, lower in males, and further declined over time, indicating that greater emphasis should be placed on attire hygiene during progression of clinical training. Notably, equipment hygiene was unsatisfactory among most medical students and appears to be a problem area with a knowledge gap which should be highlighted and addressed during clinical training. The findings of this study suggest that knowledge, attitudes and practices among medical students, particularly on equipment and attire hygiene are neglected areas which need to be addressed and emphasised throughout medical training.

## Additional file

Additional file 1: Questionnaire: Questions used to assess knowledge, attitude and practice in relation to hand, attire and equipment hygiene. (DOCX 22 kb)

## Abbreviations

AH: Attire hygiene
EH: Equipment hygiene
HH: Hand hygiene
KAP: Knowledge attitudes and practice
