# Supplementary material for: Hygiene practices during clinical training: knowledge, attitudes and practice among a cohort of South Asian Medical students
Source: BMC Med Educ. 2019 May 21;19:157. doi: 10.1186/s12909-019-1582-2 (PMC6528303; doi:10.1186/s12909-019-1582-2)
Supplement: Supplementary file 1 — Questionnaire: Questions used to assess knowledge, attitude and practice in relation to hand, attire and equipment hygiene. (DOCX 22 kb) [file 12909_2019_1582_MOESM1_ESM.docx]

Additioanl file 1: Questionnaire

**Knowledge**

**Hand Hygiene**

- It is better to wash hands with soap rather than hand rub when the hands are visibly soiled.
- It is necessary to wash hands after removal of gloves.
- It is necessary to keep fingernails short and clean.
- It is recommended to wash hands regularly.
- When washing hands, it is necessary to rub the hands together for **at least 10 seconds**.
- It is necessary to wash hands prior to an aseptic procedure.
- It is **not** necessary to wash hands after contact with body fluids.
- It is necessary to hands free from accessories
- It is necessary to wash hands after direct patient contact.
- It is necessary to wash hands with hand rub rather than soap when caring for patients with vomiting or diarrhoea.

**Hygiene related to attire**

- It is recommended to clean the clinical coat daily during clinical appointments.
- It is advisable to keep the clinical coat on, when consuming meals.
- It is better to use detergent over normal soap to clean the clinical coat regularly
- It is recommended to clean the clinical coat at least once a week during clinical appointments.
- Clinical coat and attire is known to harbour harmful pathogens

**Hygiene related to equipment**

- It is advisable to immerse the stethoscope in any liquid
- Toothpicks used for checking sensory deficits can be re-used.
- Ear tips of the stethoscope should be removed when cleaning.
- Wiping with 70% isopropyl alcohol will disinfect the stethoscope.
- It is recommended to clean the stethoscope regularly.

**Attitudes**

**Hand hygiene**

- I feel I need to wash my hands prior to an after direct patient contact.
- I feel it is necessary to rub the hands together for at least 10 seconds when hand washing
- I feel it is necessary to wash hands with soap rather than hand rub when caring for patients with vomiting or diarrhoea.
- I feel that keeping my hands free from accessories will have a significant effect on prevention of transmission of infection.
- I feel it is necessary to wash hands with soap rather than hand rub when the hands are visibly soiled.
- I feel I need to wash my hands prior to an aseptic procedure.
- I feel I need to wash my hands each time after contact with body fluids.
- I feel that keeping my fingernails short and clean will help to reduce transmission of infection.
- I feel that it is always necessary to follow the steps of hand washing correctly.
- I feel it is necessary to wash my hands after removal of gloves.

**Hygiene related to attire**

- I feel that **ideally** I need to clean my clinical coat daily during clinical appointments to prevent transmission of infection.
- I feel that cleaning my clinical coat at least once a week during clinical appointments would be beneficial to prevent transmission of infection.
- I feel that using normal soap over detergent will be beneficial to clean my clinical coat.
- I feel I should take off the clinical coat, when consuming meals.
- I feel my clinical coat and attire can harbour harmful pathogens

**Hygiene related to equipment**

- I feel that I need to wipe my stethoscope with 70% isopropyl alcohol to disinfect it **daily**.
- I feel I need to know how to clean my stethoscope.
- I feel it is necessary to clean my stethoscope and other equipment regularly.
- I feel that I need to be careful **not** to immerse my stethoscope in any liquid.
- I feel that removal of the ear tips of my stethoscope for thorough cleaning is needed.

**Practice**

**Hand Hygiene**

- I keep my fingernails short and clean.
- I wash my hands prior to direct patient contact.
- I wash my hands with soap rather than hand rub when the hands are visibly soiled
- I wash my hands prior to an aseptic procedure.
- I wash my hands after direct patient contact.
- I wash hands with soap rather than hand rub when caring for patients with vomiting or diarrhoea.
- I rub the hands together for **at least** 10 seconds when washing my hands.
- I wash my hands after removal of gloves.
- I practice the steps of hand washing.
- I wash my hands after contact with body fluids.

**Hygiene related to attire**

- I clean my clinical coat daily during clinical appointments.
- I use detergent rather than soap to clean my clinical coat.
- I clean my clinical coat at least once a week during clinical appointments.
- I consume my meals wearing the clinical coat.
- I clean my clinical attire separately.

**Hygiene related to Equipment**

- I wipe my stethoscope with 70% isopropyl alcohol to disinfect it.
- I take care **not to** immerse my stethoscope in any liquid.
- I re-use toothpicks for checking sensory deficits.
- I remove the ear tips of my stethoscope for thorough cleaning.
- I clean my other medical equipment eg: knee hammer, tape following patient contact daily.
